# “What has dentistry learned from the pandemic?”

**DOI:** 10.1007/s00784-023-05036-9

**Published:** 2023-05-10

**Authors:** Mariano Sanz

**Affiliations:** Faculty of Odontology, University Complutense of Madrid, Madrid, Spain

The emergence of the novel variant of the severe acute respiratory syndrome–related coronavirus (SARS-CoV-2) and the subsequent global coronavirus disease (COVID-19) pandemic has evolved rapidly into a public health crisis, having a significant impact on oral health systems across the world. In fact, by the end of 2022, more than 600 million people globally were confirmed COVID-19 cases, including over 6 million deaths from this disease (World Health Organization [1].

Among the major risk factors, the presence of periodontitis has been explored as a relevant comorbidity, increasing the risk both to contract the infection and to develop more severe patterns of disease [2–4]. This virus is mainly spread via respiratory droplets and aerosols from infected persons reaching the mucosa of the upper respiratory track or the oral cavity. On binding to epithelial cells in these lining mucosa, SARS-CoV-2 starts to invade the host and to replicate, causing in most of the patients an asymptomatic or mild respiratory infection [5]. However, in some individuals the viremia may cause a strong immune response in predisposed individuals, leading to an hyperinflammatory response, which may cause acute respiratory distress syndrome (ARDS) and respiratory failure, which is considered the main cause of death in patients with COVID-19 [6].

Despite the entire population being potentially susceptible to this disease, the identification of patient’s risk factors has been critical in developing efficient individualized and population-based preventive strategies. Among them, people with underlying co-morbidities including obesity, hypertension, diabetes mellitus, cardiovascular disease (CVD), chronic kidney disease, chronic obstructive pulmonary disease (COPD), asthma, immunosuppression, and cancer have been significantly associated with severe COVID-19 [7]. Among these potential co-morbidities associated with COVID-19, periodontitis has been suggested considering its high prevalence and the well-established epidemiological associations with most noncommunicable chronic diseases, particularly diabetes, CVD, obesity, and many respiratory diseases, such as pneumonia and COPD [8–10]. Besides shared genetic and environmental risk factors, these relationships have been explained through common infective/inflammatory pathways, such as bacteraemia, bacterial aspiration into the lower respiratory tract, systemic inflammation and induced autoimmune damage [11].

The implications of oral health in this pandemic viral infection go beyond its possible association with an increased severity in case of SARS-CoV-2 infection, since the oral cavity has a key role in the transmission and pathogenicity of this and other respiratory viruses, and therefore, dental care delivery with its inherent production of aerosols in the dental settings may impact this transmission. In light of these implications, the use of preprocedural antiseptic agents must be re-evaluated considering the current scientific evidence of efficacy.

This special issue reviews the current state of knowledge regarding the importance of the oral cavity in the transmission and infectivity of respiratory viral infections, with special emphasis on COVID-19 disease and the SARS-CoV-2 virus*.* The first review “*The role of the oral cavity in SARS-CoV-2 and other viral infections”* [12] assesses the role of the oral cavity in SARS-CoV-2 and other viral upper respiratory tract infections that use the oral cavity as the portal of entry and replication for infection. This review discusses on the different mechanisms used by respiratory viruses in their entrance and replication in the body through the oral cavity. It evaluates the role of the oral cavity in the diagnosis of these infections and the general measures that can be imposed in the prevention and control of these infections.

The second review *"Aerosol in the Oral Health-Care Setting: -a misty topic-"* [13] assesses the importance of aerosol transmission in the spread of viral diseases. It defines the mechanisms of action of these suspensions of liquid or solid in air that are defined by the size of the inspired particle. The review not only includes the sources of dental aerosol, but also their potential health threats defining the type of aerosol generating procedures (AGPs) regularly used in dentistry and their importance as a source of transmission to patients and oral health personnel. The paper concludes with recommendations and strategies for controlling and mitigating the possible deleterious effect of dental aerosol and spatter.

The third review “*Preprocedural mouthwashes for infection control in dentistry – an update”* [14] summarizes the clinical evidence on the use of preprocedural mouthwashes and the various antiseptic agents aimed at the control of bacteria, viruses and specifically the SARS-CoV-2. It also reviews the preventive effect of using preprocedural mouthwashes with antibacterial and virucidal action, such as cetylpyridinium chloride (CPC), to temporarily reduce the bacterial or viral burden in the oral cavity during the provision of dental procedures and therefore, to mitigate and control the possible effect of dental aerosols. It also discusses their possible side effects and potential risks in their extended chronic use.

Finally, the fourth review: “*Oral and systemic health: is there a new link with COVID-19* [15]*?* evaluates the evidence behind the association between periodontitis and COVID-19 severity based on observational studies. This review not only evaluates the epidemiological evidence but also the biological plausibility of these associations with special emphasis on the impact of the significant bacteraemia and systemic inflammation that occurs in severe periodontitis patients and by the possible sharing of common genetic or environmental risk factors.

In conclusion, this special issue provides updated relevant information not only on what we have learnt from the COVID-19 infection and the implications of oral health in the spread and severity of this infection, but also on how the implementation of simple preventive measures such as preprocedural mouth rinsing can provide a safe environment for oral health practice, even amid a global viral pandemic.
